# Combined leadless pacing and subcutaneous defibrillation strategy in a high-risk patient: first case report from Peru

**DOI:** 10.47487/apcyccv.v6i4.513

**Published:** 2025-12-29

**Authors:** Alexis Vallejos-Barrientos, Diego Davila-Flores, Richard Soto-Becerra, Mario Cabrera-Saldaña, Carolina Guevara-Caicedo, Ana Cecilia Gonzales-Luna, Ángel Cueva-Parra, Marisel Payano-Rojas, Pío Zelaya-Castro

**Affiliations:** 1 National Cardiovascular Institute-INCOR, EsSalud, Lima, Peru. National Cardiovascular Institute-INCOR EsSalud Lima Peru; 2 Arrhythmia Unit, National Cardiovascular Institute-INCOR, EsSalud, Lima, Peru. Arrhythmia Unit National Cardiovascular Institute-INCOR EsSalud Lima Peru

**Keywords:** Pacemaker, Artificial, Defibrillators, Heart Failure, Infection, Marcapaso Artificial, Desfibriladores Implantables, Insuficiencia Cardíaca, Infecciones

## Abstract

We present the case of a 51-year-old male with non-ischemic dilated cardiomyopathy and complete atrioventricular block, who was previously implanted with a cardiac resynchronization therapy defibrillator. The patient developed signs of pocket infection with a high risk of extrusion. Partial system extraction was performed, followed by 14 days of intravenous antibiotic therapy. Due to a history of ventricular fibrillation and permanent pacing dependency, and in the absence of viable transvenous access, a sequential implantation strategy was adopted using a leadless pacemaker (Micra AV, Medtronic) and a subcutaneous implantable cardioverter-defibrillator (EMBLEM, Boston Scientific). Both procedures were completed without complications, and the patient showed favorable recovery, with effective pacing, no arrhythmic recurrences, and no signs of infection at the six-month follow-up. This case illustrates the feasibility of a fully leadless approach in high-risk patients with contraindications to conventional transvenous systems.

## RESUMEN

Presentamos el caso de un varón de 51 años con miocardiopatía dilatada no isquémica y bloqueo auriculoventricular completo, previamente portador de un desfibrilador con terapia de resincronización cardíaca. El paciente desarrolló signos de infección del bolsillo del dispositivo con alto riesgo de extrusión. Se realizó una extracción parcial del sistema, seguida de 14 días de terapia antibiótica intravenosa. Debido a los antecedentes de fibrilación ventricular, dependencia de estimulación permanente y ausencia de acceso transvenoso viable, se optó por una estrategia de implante secuencial con un marcapasos sin cables (Micra AV, Medtronic) y un desfibrilador subcutáneo (EMBLEM, Boston Scientific). Ambos procedimientos se realizaron sin complicaciones, y el paciente mostró una recuperación favorable, con estimulación efectiva, sin recurrencias arrítmicas ni signos de infección al sexto mes de seguimiento. Este caso demuestra la factibilidad de una estrategia completamente libre de cables en pacientes de alto riesgo con contraindicación para sistemas transvenosos convencionales.

## Introduction

Fully leadless cardiac systems, combining a leadless pacemaker (LP) and a subcutaneous implantable cardioverter-defibrillator (S-ICD), offer a valuable alternative for patients with contraindications to transvenous devices. ^1^ LPs are indicated for patients requiring single-chamber ventricular pacing, particularly when venous access is compromised or the risk of infection is high. ^2^^,^^3^ S-ICDs are recommended for the prevention of sudden cardiac death in patients who do not need bradycardia or anti-tachycardia pacing. ^4^^,^^5^

Although early data suggest this combined approach is feasible and safe, real-world experience remains limited, particularly in Latin America. ^6^^,^^7^ We report the first documented case in Peru of sequential implantation of an LP and an S-ICD in a patient with a prior cardiac resynchronization therapy defibrillator (CRT-D) infection, complete atrioventricular block, and a history of ventricular fibrillation. This case illustrates the role of fully leadless systems in complex device management and reinforces their potential in selected high-risk patients.

## Case report

A 51-year-old male with hypertension and non-ischemic dilated cardiomyopathy (initial left ventricular ejection fraction: 15%) underwent ICD implantation for primary prevention in 2016. During follow-up, an upgrade to CRT-D was indicated and performed via the right deltopectoral approach due to left subclavian vein stenosis. A complete atrioventricular block was documented during the procedure, necessitating the use of permanent ventricular pacing. The patient continued on optimal medical therapy for heart failure, consisting of bisoprolol, spironolactone, and enalapril, as per the prevailing guidelines at that time.

During the first four years following CRT-D implantation, the patient was considered a non-responder because he did not meet the established response criteria: ^1^ electrocardiographic narrowing of the QRS complex by more than 20% from baseline; ^2^ echocardiographic evidence of reverse remodeling, such as a reduction in left ventricular end-systolic volume by more than 10% or an improvement in LVEF by more than 5%; ^3^ quality-of-life improvements, including an increase of at least 50 meters in the six-minute walk test, at least a 1 mL/kg/min increase in peak oxygen consumption, or a 20-30 point reduction in the Minnesota Living with Heart Failure Questionnaire; and ^4^ a reduction in heart failure-related hospitalizations. Subsequently, after the addition of guideline-directed medical therapy with sacubitril/valsartan, dapagliflozin, and amiodarone, the patient showed marked clinical and echocardiographic improvement (NYHA class II, LVEF: 48%).

**Figure 1:**
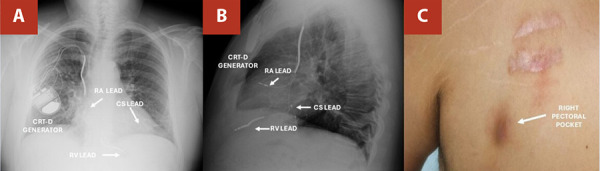
Initial evaluation (**A**, **B**, arrows) Posteroanterior and lateral chest radiographs show a right pectoral cardiac resynchronization therapy defibrillator (CRT-D) generator with active fixation leads in the right atrium (RA) and right ventricle (RV) and a passive fixation lead in the coronary sinus (CS). (**C**, arrows) Right pectoral CRT-D generator pocket with local signs of infection.

CRT-D: Cardiac resynchronization therapy defibrillator; RA: Right atrium; RV: Right ventricle; CS: Coronary sinus.

In late 2024, the patient arrived to our institution with one-month history of localized skin changes at the generator pocket. Examination revealed a violaceous 4x2 cm lesion with skin thinning over the generator site, though he was afebrile. Laboratory results showed mild leukocytosis (11.8 x 10^9^/L), elevated C-reactive protein (10.9 mg/L), normal procalcitonin, and negative blood cultures. Transesophageal echocardiography excluded vegetation. A diagnosis of pocket infection with a high risk of device extrusion was established. (Figure 1)

We initiated empirical intravenous vancomycin (1 g every 8 hours) and ciprofloxacin (400 mg every 12 hours). Surgical intervention included complete removal of the generator and the coronary sinus lead and partial removal of the right ventricular lead due to significant fibrosis. A temporary active-fixation pacing system was placed during the 14-day antibiotic regimen.

A multidisciplinary team determined that the patient was a cardiac resynchronization therapy non-responder with a secondary prevention indication for an implantable cardioverter-defibrillator and permanent pacing due to a complete atrioventricular block. Given left subclavian stenosis, suspected active infection, and risk of re-infection from abandoned leads, transvenous reimplantation was ruled out. A completely leadless approach was selected, involving the sequential implantation of an LP and an S-ICD.

**Figure 2:**
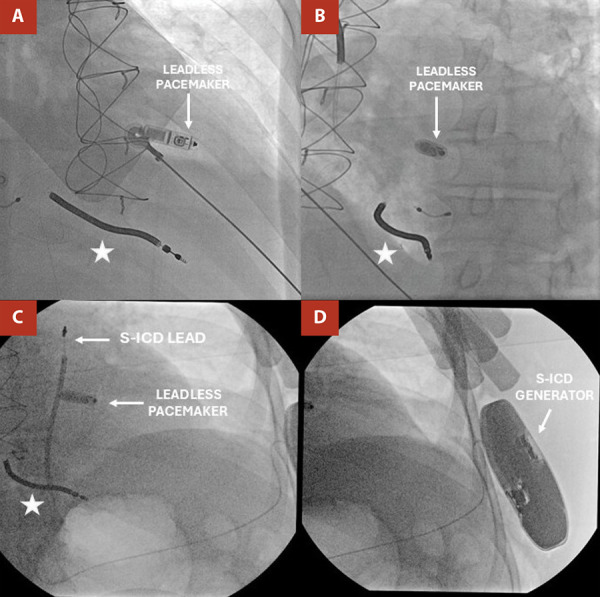
Leadless pacemaker implantation. (**A**, arrows) Fluoroscopy in right anterior oblique projection showing the leadless pacemaker implanted in the high mid-septal region of the RV. (**B**) Fluoroscopy in left anterior oblique projection confirms leadless pacemaker placement in the same septal region. S-ICD implantation. (**C**, arrows) Fluoroscopy image showing the S-ICD lead positioned at the third left parasternal ICS and the leadless pacemaker implanted in the mid-septal region of the RV. (**D**) Fluoroscopy image showing the S-ICD generator located at the 5th left ICS.

Under general anesthesia and fluoroscopic guidance, a single-chamber Micra TPS AV (Medtronic) was implanted via the right femoral vein. An 8Fr sheath and a 0.034 Amplatz guidewire were used. The 23Fr Micra introducer was positioned in the right atrium. The delivery system advanced the device through the tricuspid ring, placing it on the high mid-septum with excellent electrical parameters (R wave: 11.9 mV; impedance: 750 ohms; threshold: 0.3 V/0.24 ms). The LP was programmed in VDD mode after manual optimization of atrial mechanical sensing, with the A4 threshold adjusted to ensure reliable atrial detection and optimal atrioventricular synchrony. Final programming: VDD 60 bpm, output 2.5V/0.24 ms. (Figure 2)

(*) Abandoned lead in the right ventricle. S-ICD: Subcutaneous implantable cardioverter-defibrillator; ICS: Intercostal space; RV: Right ventricle.

Four days later, the patient passed the S-ICD screening test in all vectors (primary, secondary, and alternate). This test assesses R-wave amplitude and stability, as well as the R-to-T wave ratio, to evaluate the risk of T-wave oversensing. An EMBLEM S-ICD (Boston Scientific) was implanted under general anesthesia. A pocket was created between the latissimus dorsi and serratus anterior muscles at the level of the fifth intercostal space in the left anterior axillary line. The lead was tunneled to the subxiphoid region and anchored in the left parasternal line (3rd intercostal space). Appropriate sensing was achieved in primary and secondary vectors. Ventricular fibrillation was induced, promptly detected by the S-ICD, and successfully defibrillated, with no interference observed in LP pacing or sensing. Final programming: detection zone 200-220 bpm; shock impedance: 120 ohms. (Figure 2)

**Figure 3:**
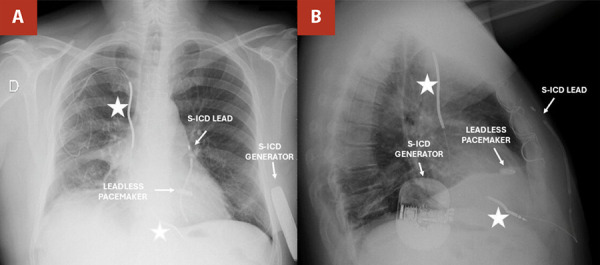
One-month post-discharge evaluation. (**A, B**, arrows) Posteroanterior and lateral chest X-ray: S-ICD generator located at the level of the fifth intercostal space (ICS), with the lead position at the 3rd left parasternal ICS; leadless pacemaker visible at the mid-septal region of the right ventricle (RV).

A chest X-ray confirmed the optimal placement of both devices. The patient was discharged on postoperative day 2 with oral antibiotics (amoxicillin/clavulanate) for 5 days, a compression belt for 30 days, and continued heart failure therapy. At one-month follow-up, he remained asymptomatic, with stable pacing, no arrhythmic events, and no signs of infection. (Figure 3)

(*) Abandoned leads in the right atrium (RA) and right ventricle (RV). S-ICD: Subcutaneous implantable cardioverter-defibrillator; RA: Right atrium; RV: Right ventricle.

**Figure 4:**
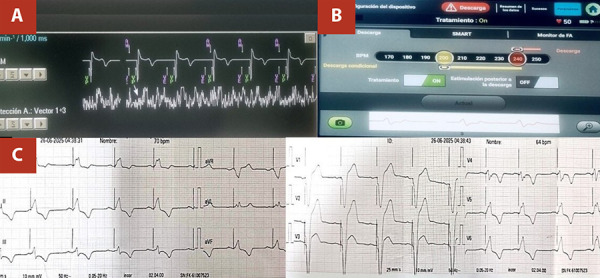
Telemetry at 6-month follow-up. **(A)** Leadless pacemaker (Micra AV, Medtronic) programmed in VDD mode (60-105 bpm), demonstrating effective atrioventricular synchrony (AM-VP: 75%) and stable electrical parameters: impedance 710 ohms, threshold 0.50 V at 0.24 ms, and R-wave amplitude 13.5 mV. **(B)** Subcutaneous ICD (EMBLEM, Boston Scientific) with no detected or treated arrhythmias. Detection zones: conditional (200-240 bpm) and shock (>240 bpm). Impedance: 85 ohms; battery longevity: 96%. **(C)** Electrocardiogram reveals sinus P waves consistently followed by ventricular paced complexes. Notably, no interference is observed between the LP and S-ICD during pacing.

LP: Leadless pacemaker; S-ICD: Subcutaneous implantable cardioverter-defibrillator; AM: Atrial mechanical sensing;

VE: Ventricular end; VP: Ventricular pacing; VS: Ventricular sensing.

At six-month follow-up, the patient remained asymptomatic, without signs of infection or arrhythmia. Device interrogation confirmed stable function of both the LP and S-ICD (Figure 4). Following the loss of cardiac resynchronization therapy, serial clinical and echocardiographic assessments showed no deterioration in heart failure status, with LVEF 48% and NYHA functional class I.

## Discussion

We report the successful sequential implantation of a leadless pacemaker (Micra AV, Medtronic) and a subcutaneous implantable cardioverter-defibrillator (EMBLEM, Boston) in a patient with a prior CRT-D infection, a permanent pacing requirement due to complete atrioventricular block, and an absence of viable venous access. This case highlights a fully leadless strategy as a viable alternative in selected high-risk patients, particularly when transvenous systems are contraindicated due to infection and vascular occlusion.

In cardiovascular implantable electronic device infections, complete system removal is the gold standard, as retained material increases the risk of recurrence. ^8^^,^^9^ In this case, complete extraction of the right ventricular lead was not feasible due to dense fibrosis after eight years, leaving a 6 cm fragment. The decision for partial removal was based on the high risk of vascular or myocardial injury. After surgery and targeted antibiotics, the patient improved clinically, remained afebrile with negative blood cultures, and showed no echocardiographic evidence of vegetations during six months of follow-up. This underscores the need for individualized strategies when complete extraction poses excessive risk. ^8^^,^^9^

LPs were developed to mitigate risks associated with transvenous systems, including lead-related complications, pneumothorax, and pocket infections. ^2^^,^^3^ Clinical studies and real-world data have consistently demonstrated a reduction in infection rates with LP over time. ^2^^,^^3^ Limitations include a lack of atrioventricular synchrony in some models, non-physiological pacing, and challenges in retrieving the system. Nonetheless, the current HRS consensus supports their use in infection scenarios with no vascular access. ^10^

S-ICDs are recommended for patients without pacing or ATP requirements, and their use in combination with LPs has been increasing. ^10^^,^^11^ However, safety concerns persist, particularly related to the oversensing of pacing spikes, which are primarily associated with unipolar pacing systems ^11^^,^^12^. In our case, the use of a bipolar Micra AV device mitigated this concern. Recent case reports, such as those by Calvagna *et al.*^11^, Mitacchione, ^13^ and Milaras *et al.*, ^14^ confirm that co-implantation of LP and S-ICD is both feasible and safe, with low rates of inappropriate shocks.

Our patient had a prior appropriate shock for ventricular fibrillation, making him a candidate for secondary prevention. While the absence of ATP capability in S-ICDs is a limitation, it is not critical in ventricular fibrillation-only scenarios such as this one. ^3^^,^^5^^,^^10^ Additionally, concerns regarding LP performance after S-ICD shocks are theoretical; the current case series suggests no negative impact on pacing thresholds or function. ^11^^,^^14^

The concept of combining a leadless pacemaker and an S-ICD was first introduced in clinical practice by Mondésert *et al.* in 2015 ^15^, who reported the first-in-human case of such a strategy following the extraction of an infected transvenous system. Their report demonstrated the feasibility of a completely leadless approach in patients with a high risk of infection. Subsequently, Tjong *et al.* published a single-center experience evaluating the safety and practicality of this dual therapy, confirming favorable outcomes in a broader cohort. ^16^ These findings laid the groundwork for further adoption of this approach in selected patient populations.

Although combined leadless device implantation is not yet incorporated into formal guideline recommendations, increasing evidence supports its use in highly selected patients. ^5^^,^^10^ This case contributes to the limited Latin American experience and reinforces the practicality of a fully leadless strategy in patients with infection and venous occlusion.

Limitations of this case include the short follow-up period and absence of S-ICD testing under LP pacing stress conditions. In conclusion, this case illustrates the feasibility and short-term efficacy of a fully leadless device strategy in selected patients. Further research is warranted to assess long-term safety and device-device interaction.
